# Communication and information sharing with pediatric patients including refugees with advanced cancer, their caregivers, and health care professionals in Jordan: a qualitative study

**DOI:** 10.3389/fonc.2023.1118992

**Published:** 2023-04-27

**Authors:** Ghadeer Alarjeh, Sabah Boufkhed, Waleed Alrjoub, Ping Guo, Sema Yurduşen, Fahad Ahmed, Mousa Abdal-Rahman, Sawsan Alajarmeh, Anwar Alnassan, Shireen Al-Awady, Tezer Kutluk, Richard Harding, Omar Shamieh

**Affiliations:** ^1^ Center for Palliative & Cancer Care in Conflict (CPCCC), King Hussein Cancer Center (KHCC), Amman, Jordan; ^2^ Department of Palliative care, King Hussein Cancer Center (KHCC), Amman, Jordan; ^3^ Cicely Saunders Institute of Palliative Care, Policy and Rehabilitation, Florence Nightingale Faculty of Nursing, Midwifery and Palliative Care, King’s College London, London, United Kingdom; ^4^ Humanitarian and Conflict Response Institute, The University of Manchester, Manchester, United Kingdom; ^5^ School of Nursing and Midwifery, Institute of Clinical Sciences, College of Medical and Dental Sciences, University of Birmingham, Birmingham, United Kingdom; ^6^ Department of Pediatric Oncology, Hacettepe University Faculty of Medicine and Cancer Institute, Ankara, Türkiye; ^7^ Department of Psychology, Ankara Science University, Ankara, Türkiye; ^8^ Department of Public Health, Faculty of Medicine, Ankara Yıldırım Beyazıt University, Ankara, Türkiye; ^9^ Department of Pediatric Oncology, King Hussein Cancer Center, Amman, Jordan; ^10^ College of Medicine, the University of Jordan, Amman, Jordan

**Keywords:** pediatric, palliative care, cancer, refugees, communication, information sharing

## Abstract

**Introduction:**

Effective communication in pediatric palliative cancer care is an important aspect of practice to enhance patient- and family-centered care, and to optimize decision-making. However, little is known about communication preferences practices from the perspectives of children, caregivers and their health care professionals (HCPs) in the Middle Eastern region. Furthermore, involving children in research is crucial but limited. This study aimed to describe the communication and information-sharing preferences and practices of children with advanced cancer, their caregivers, and health care professionals in Jordan.

**Methods:**

A qualitative cross-sectional study was conducted using semi-structured face to face interviews with three groups of stakeholders (children, caregivers and HCPs). Purposive sampling recruited a diverse sample from inpatient and outpatient settings at a tertiary cancer center in Jordan. Procedures were in line with the Consolidated criteria for reporting qualitative research (COREQ) reporting guidelines. Verbatim transcripts were thematically analyzed.

**Findings:**

Fifty-two stakeholders participated: 43 Jordanian and 9 refugees (25 children, 15 caregivers and 12 HCPs). Four major themes emerged: 1) Hiding information between the three stakeholders which includes parents who hide the information from their sick children and ask the HCPs to do so to protect the child from distressful emotions and children who hide their suffering from their parents to protect them from being sad; 2) Communication and sharing of clinical versus non-clinical information; 3) Preferred communication styles such as use of a companionate approach by acknowledging patients and caregivers’ suffering, building a trustful relationship, proactive information sharing, considering child age and medical status, parents as facilitators in communication, and patients’ and caregivers’ health literacy; 4) Communication and information sharing with refugees where they had dialect issues, which hindered effective communication. Some refugees had unrealistically high expectations regarding their child’s care and prognosis, which posed challenges to communication with staff.

**Discussion:**

The novel findings of this study should inform better child-centered practices and better engage them in their care decisions. This study has demonstrated children’s ability to engage in primary research and to express preferences, and parents’ ability to share views on this sensitive topic.

## 1 Introduction

Around 300,000 cases of children worldwide are diagnosed with cancer each year (1, 2). Although around 80% of those patients are cured in high income countries, this drops to 20% in low- and middle-income countries (3). Cancer is a leading causes of death among children (4), and will become the leading cause of serious health-related suffering for any age at the end of life globally (5). In the Eastern Mediterranean region, cancer is the third leading cause of death for children aged 5-14 years (6). In 2015, nearly 245 new Jordanian pediatric patients were diagnosed with cancer, and it was estimated that about 62 new pediatric Syrian refugee patients in Jordan were diagnosed with cancer (6).

Tailoring effective communication according to cancer patients’ preferences and needs, during cancer treatment trajectory, is considered as a crucial component of patients’ centered care. It was correlated with patient adherence to treatment, decision making, and satisfaction with care and had shown a noticeable impact on patients’ quality of life (7). Effective communication between patients, family and HCPs requires a humanitarian touch, compassionate connection, interdisciplinary team collaboration, and coordination and follow up to meet patients, family, and HCPs needs (8).

Palliative care is now established as an essential health service within Universal Health Coverage. Effective communication and information is essential to sharing information about diagnosis and prognosis and for informed decision-making when cure is unlikely. Good communication can improve children’s health related outcomes positively (2, 9, 10), enable children to better cope better with their disease, treatment, end-of-life care and relapse (11, 12) and for the families when curative therapy is unsuccessful and families are grieving (8).

Evidence to date regarding communication and information sharing in pediatric palliative care settings has mainly originated in Western cultural settings (13). The existing evidence in this field has focused on parents sharing cancer diagnoses with their children (14), and adolescents’ experience of their diagnosis (15). The pediatric palliative care research literature has not recruited child refugees (16). Similar research among adult refugees with advanced cancer revealed specific issues relating to compounded trauma and fractured social networks (17).

This study aimed to describe the communication and information-sharing preferences and practices of children with advanced cancer, their caregivers, and health care professionals in Jordan.

## 2 Materials and methods

### 2.1 Study design and settings

We conducted a qualitative cross-sectional study with semi-structured face-to-face interviews. It was conducted within inpatient and outpatient clinics of a tertiary cancer center serving >60% of adult and pediatric Jordanian patients with cancer and patients from surrounding countries (18).

GA, WA and SA (senior researchers and instructors at adult palliative care department) conducted the Jordanian interviews with three groups of stakeholders (children, caregivers, and HCPs). The consolidated criteria for reporting qualitative research (COREQ) guidelines were followed (19). The research questions were: 1) What are their communication and information sharing practices and preferences? 2) What are the communication challenges in this population and what might be the best practice? Primary data for these questions was collected to improve child- and family-centered care and palliative care through local health policies, clinical guidelines, and local practices in Jordan and regionally.

### 2.2 Research study participants

Inclusion/exclusion criteria for each population were as follows:

Children and young people (hereafter “children”) aged from 5-18 years with advanced cancer (Stage III or IV as determined by their treating clinicians) who had been seen by palliative care team at least once. Parents or main caregivers of children who met the inclusion criteria.

Children or caregivers who were unable to communicate, to speak Arabic or English, or those were unable clinically to provide assent/consent (written and/or verbal) were excluded.

Health care professionals (HCP) with at least 6 months’ clinical experience of managing children with advanced cancer (doctors, psychologists, social workers, child life specialists and nurses).

We purposively sampled the populations by gender, age, child diagnosis and HCP profession. The aim was to recruit participants till reaching data saturation with respect to the study aim.

### 2.3 Recruitment and ethical considerations

Eligible participants were identified and approached by one of the three clinically experienced trained Jordanian researchers (GA, SA and WA) with the support of the pediatric palliative care nurse coordinator (SA). Each population sample was recruited and interviewed using a customized interview topic guide, information sheet and consent form. Furthermore, for children the topic guides, information sheets and assent forms were designed appropriately for their age and developmental stage (5-7, 8-10, 11-16 and 17-18 years, in line with ongoing research in this population (20–22).

If the participant agreed to participate, they were asked to sign a consent form. An assent form was also obtained from children 5-18 years old following their parents’ approval (23). A distress protocol was used to reduce potential harms (24).

Ethical approval was obtained from both: Kings College London (Study Reference: HR-18/19-8838) and King Hussein Cancer Centre (Study Reference: 18 KHCC 162) prior to any study recruitment.

### 2.4 Data collection

GA, WA and SA conducted and digitally audio recorded the interviews in Arabic language using appropriate topic guide (decided with the family). Interviews were conducted at a private convenient place and time, after obtaining informed consent/assent forms from the participants. However, due to COVID-19 restrictions, five interviews were conducted by telephone following further institutional review board approvals.

The interviews were carried out between April 23rd 2019 and July 29th 2020. Qualitative methods training workshops were held with local research team (led by RH PG OS). The interviewers had no previous knowledge or relationships with any participants prior to the consenting or interviewing process. The main caregivers who participated in this study were not related to any recruited child participant.

The interview topic guides were developed by the core project team members following review of relevant literature. The interviewers started the interview by building a rapport with the participants, then used the topic guide’s open-ended questions. For example, they played and drew with children before and during the interviews to encourage them to interact, to answer the topic guide questions, and to complete the interviews effectively. The questions mainly focused on children’s concerns, needs, preferences and challenges in relation to the four palliative care domains of physical, social, psychological, and spiritual (25).

Before the end of each interview, the interviewer summarized the information back to participants for clarification and any further data was added to enhance data credibility (24). The majority (n = 23) of pediatric patients (especially those 15 years old or below) were accompanied by their caregivers during the interviews, and the caregivers were permitted to assist the child in the interview when needed.

### 2.5 Data analysis

Descriptive analysis of sample characteristics was followed by two local researchers (GA and WA) transcribing interviews verbatim. Transcriptions were pseudonymized, translated into English language by two local professional translators, then the two local researchers did a data quality check on the translation for accuracy, then imported to NVIVO 12 PRO software. The joint research team (Jordan and UK) applied the six phases of inductive thematic coding (**Table 1**). Frequent virtual meetings were held, and the thematic framework was refined during an iterative process by rearranging, adding, and deleting some generated themes and codes after discussion and agreement of the full team (26). The proposed coding framework was then presented to the wider research team for review, refinement and approval. The final approved framework was used by (GA and SB) to analyze the whole data after discussion and agreements of the teams on the findings’ interpretations.

**Table 1:** Six phases of thematic analysis.

Phase 1: familiarization process	• ‘repeated reading’ of the interview transcripts before analysis • Transcription of the recoded interviews by GA and WA
Phase 2: developing initial codes	• Initial coding developed by the research team members (SB, GA, WA, FA, SY). • Frequent virtual meetings were done, and the codes was refined after discussion and agreement of the involved research team members
Phase 3: identifying themes	• The codes and the coded data extracts were sorted and collated into possible selected broader themes and GA developed the initial thematic map
Phase 4: themes refinement	• Themes were reviewed by the involved research team members for possible modification. Some themes were deleted, merged into one theme, or one theme have different ideas that had been divided into more themes.
Phase 5: themes explanation and naming	• Each theme was refined by taking into consideration the overall picture to be clearly defined and named.
Phase 6: generating final report	• writing up the finalized research report using analytical narrative approach that was supported by representative quotes taken form the participants interview transcripts

## 3 Results

### 3.1 Participant characteristics

52 interviews were conducted. Of those, 43 were Jordanian and 9 were refugees. 100% of those who were approached were recruited. Their demographics are reported in **Table 2**.

**Table 2:** Participant characteristics (N=52).

	Children		Caregivers		HCPs	
	n	%	n	%	n	%
Gender:						
Female	14	56	12	80	9	75
Male	11	44	3	20	3	25
Nationality						
Jordanian	21	84	10	67	12	100
Libyan	1	4	2	13	N/A	
Palestinian	1	4	2	13	N/A	
Syrian	2	16	1	7	N/A	
Children diagnosis classifications						
i. Leukaemia, myeloproliferative diseases, and myelodysplastic diseases.	6	24	5	33	N/A	
ii. Lymphomas and reticuloendothelial neoplasms.	2	8	1	6	N/A	
iii. CNS and miscellaneous intracranial and intraspinal neoplasms.	1	4	3	20	N/A	
iv. Neuroblastoma and other peripheral nervous cell tumours.	0	0	4	26	N/A	
v. Renal tumors.	1	4	1	6	N/A	
vi. Malignant bone tumors.	12	48	0	0	N/A	
vii. Soft tissue and other extraosseous sarcomas.	2	8	1	6	N/A	
viii. Other malignant epithelial neoplasms and malignant melanomas.	1	4	0	0	N/A	
Relationship with children:						
Mother	N/A		11	73		N/A
Father	N/A		3	20		N/A
Grandmother	N/A		1	7		N/A
Healthcare profession:						
Medical staff	N/A		N/A		7	58
Non-medical staff	N/A		N/A		5	42

HCPs, Health care professionals; N/A, Not Applicable, Children diagnosis classification using the International “Classification of Childhood Cancer, third edition: Main Classification Table”. Source: (27, p. 1459) **Table 1**, Medical staff: Nurses and doctors, Non-medical staff: Social workers, psychologist, and child life specialists.

Median duration of interviews (minutes) was as follows: children=28.5 (range=16-62); caregivers=39.5 (range=27-80); HCP=50 (range=37-116).

### 3.2 Main findings

Four major themes emerged: 1) Hiding information between the three stakeholders; 2) Communication and sharing of clinical (physical) versus non-clinical (social, spiritual, and psychological) information; 3) Preferred communication styles such as use of a companionate approach by acknowledging patients and caregivers’ suffering, building a trustful relationship, proactive information sharing, considering child age and medical status, parents as facilitators in communication, and patients’ and caregivers’ health literacy; 4) Communication and information sharing with refugees where they had dialect issues, which hindered effective communication (**Table 3**). Themes were supported by quotes with basic demographic description.

**Table 3:** Communication and information sharing themes and sub-themes.

Themes	Sub-themes
**1. Hiding information between the three stakeholders (Children, HCPs and parents)**	**1.1 Parental gatekeeping.** • Parents and HCPs, upon parents’ request, shared that they hide bad news from the child. **1.2 Children hiding information from their families.** • Children hide their suffering from their families to protect them from feeling sad. **1.3 Hiding information, trust, and treatment compliance.** • Hiding information from children may affect their trust on others and may interfere with their compliance with treatment.
**2. Communication and sharing clinical versus non-clinical information**	**2.1 Sharing adequate clinical information by the HCPs** for caregivers and children. **2.2 Stakeholders lack awareness of non-clinical issues.** • HCPs are not involved with children and family’s non-clinical information. • The three stakeholders are sharing clinical information more than non-clinical ones. **2.3 Communication in other than physical domain is helpful.** Helps in: • venting. • better understanding patients’ needs. • better assessment of child’s physical issues.
**3. Preferred communication styles**	**3.1 Preferred way of communication that HCPs need to use when communicating with caregivers and children** • Compassionate and understanding way. • Active listening. **3.2 Effective communication needs a trustful relationship between the three stakeholders** • Trustful relationship need time, experienced staff, and honesty. **3.3 Proactive information sharing by HCPs** • Helps in decreasing the level of uncertainty, better coping and enhancing the feeling of reassurance. • Caregivers want to be proactively informed **3.4 Caregivers and children need for more information/health literacy** • **Health literacy** of children and caregivers need to be taken into consideration during communication and information sharing process because some children and caregivers, with limited health literacy, may need more information to understand things better. **3.5 Collaboration and coordination between staff** • Needed to enhance effective communication • Can be done directly or indirectly though meetings and over phones. • Regular meetings are recommended • Lack of communication and coordination between the staff can affect patients’ care. **3.6 Age and developmental stage** • Adapting communication to children ages and developmental stages. **3.7 Medical status** of children should be taken into consideration when communicating with them to ensure effective communication and avoid conflicts. **3.8** According to some HCPs and few caregivers, **caregivers understand their child needs and complains better than HCPs**. • Caregivers/parents can help HCPs in better assessment of the child especially younger ages and those with certain communication limitations. • Few parents cannot understand their child’s needs and complaints.
**4. Communication and information sharing with refugees**	**4.1 Refugees reported similar communication practices, issues and needs like Jordanian** **4.2 Language, dialect issues, and discrimination** • Refugees reported no language barrier and no feeling of discrimination. • Some HCPs and refugees had dialect issues, which hindered effective communication. **4.3 High expectations** • Some refugees had unrealistically high expectations regarding their child’s care and prognosis, which posed challenges to communication with staff.

#### 3.2.1 Hiding information between the three stakeholders

The three stakeholders shared that they did not share all information with all the other partners, and hide some information from each other. Interestingly, no interviewee mentioned that parents may be hiding the information from the healthcare workers.

Parents and HCPs, upon parents’ request, shared they mainly hide bad news, such as cancer diagnosis and bad prognosis, from the sick child to protect them from feeling bad.

“The next day he told my father, and my parents kept it from me, they kept telling me it was a microbe in the blood and not to be scared. They told me my treatment would take a while, when I was admitted here, they knew I didn’t know it was leukaemia.” (child, age 16, male, Jordanian)

“We need the parents’ permission to know to what extent we can speak because you are not allowed to talk about everything with the child, or they don’t want the child to know that he is in a critical situation and he’s near death even though the child usually knows.” (Medical HCP, female, 10 years of experience).

Furthermore, some parents stated that they were not sharing the information with their young children because they thought that such young children cannot understand cancer related information.

“I think they start understanding at the age of 9. My nephew is eight and a half years old, he understands things now, he asks me questions about my daughter, but I don’t think younger children can understand. One of our relatives, who is friends with my daughter, is seven years old, and she doesn’t understand what cancer is” (Mother of a 3 years old child, age 35, Jordanian)

Some HCPs also reported challenges in sharing information with both children and parents during the same encounter. Staff were often asked by the caregivers to hide certain information (mainly negative information such as bad prognosis or relapse) from the child. HCPs reported they would then ask the child to go out the meeting room or rearrange another meeting with the caregivers alone when they wanted to share negative information. This attitude may affect HCPs’ relationship with the child, especially with those who want to know more about their health status.

“A lot of the time, especially if we have the father and mother, both are present, so we take the- yeah go to the playroom, go dispense the medicine [ … ] Sometimes if it’s not possible or the child is asking why we are speaking to the parents alone, we will call the parents for a meeting outside the clinic on a different day if it’s possible.” (Medical HCP, female, 10 years of experience).

On the other hand, some children also hide information from their parents. They tended to hide their suffering and symptoms from their families to protect them from feeling sad. So, HCPs develop strategies to navigate the conflicting demands and communication needs by going back to see some children when their family are not around.

“Because sometimes a child can’t talk to just anyone, especially his parents. When I talked to you earlier, I felt relieved, but I can’t go and talk to my dad, and I can’t tell mom everything. If I was to talk to my siblings about it, it would make them sad, and I don’t like seeing them sad.” (child, age 15, female, Jordanian)

“We would ask if there is anything about the child; and the mother “no, there is nothing”, and I always tried to go back, especially if the patient was inpatient, I tried to find a chance, when the mother is praying or is out to get something, to sit with the boy or the girl and ask them the questions I needed.” (Medical HCP, female, 15 years of experience)

Arranging for these additional meetings with parents alone or the child requires extra time and effort for services that are already busy.

“I think it is too much for him. Dr. X clinic is every Tuesday, he is busy with his clinic patients, and I don’t know how much time he has to check on his inpatients” (Mother of a 3 years old child, age 35, Jordanian)

Hiding information from children, especially, teenagers may affect their trust in their parents. They may become doubtful and lose trust in their parents and HCPs which may interfere with their compliance with treatment.

“But there are the older ones that we consider teenagers those start to lose trust in their parents because they kept [information] from them.” (Non-Medical HCP, female, 3 years of experience)

“For example, some young children around the age of 8 and 9 can read what is written on the medication or on the badges and go and Google it. When they figure out what it’s for and that they have cancer, they stubbornly refuse to continue treatment – and we have a couple of cases like this. They become so upset from their parents for hiding it from them, they become skeptical of them and think about what else they’ve been hiding from them. They refuse treatment.” (Non-Medical HCP, female, 7 years of experience)

Few HCPs stated that some parents make decisions regarding their children care plans without involving or informing the child, which was also confirmed by some children.

“They offered to amputate my leg from the hip down. My parents refused, but they didn’t tell me about it.” (child, age 17, male, Jordanian)

Some children reported being fine with certain information being hidden from them. They preferred that medical HCPs talked to their parents first. They thought that this was helpful and more convenient for them, especially when sharing bad news.

“I came here and started getting treatment for six months and I didn’t know I had cancer. I owe all of this to my parents, it was a very helpful step … I still thank them for it, I tell them it’s the best step they’ve done with me.” (child, age 16, male, Jordanian)

“No, my mom is the one who used to talk to [the doctor], she shouldn’t say anything [to me] if it’s not good.” (child, age 15, female, Jordanian)

However, nearly half of the interviewed children said that they wanted to know more about their illness.

“When they are talking at the door, I wish I can be a mosquito so I can know what they are saying.” (child, age 14, female, Syrian)

“Interviewer: Would you like if they came and spoke to you? should they come and tell you or speak to your mom and dad first? child: Sometimes they don’t tell me. Interviewer: Sometimes they don’t tell you. You would like if they can tell you? child: Yes, just for me to know” (child, age 8, female, Jordanian)

#### 3.2.2 Communication and sharing clinical versus non-clinical information

Many caregivers and some children shared that they needed HCPs to share enough information especially regarding treatment plan, disease prognosis and side effects, and that their needs for information were satisfied.

“They [The HCPs] always provided me with all the full information, and they’d tell me what’s happening, what the plan is for the future, and what the possibilities are.” (Mother of a 3 years old child, age 26, Jordanian)

In contrast, few parents stated that they did not receive enough clinical information and wanted to know more, especially about prognosis and future plans. They mentioned that hiding information such as disease progression could cause conflicts with HCPs and increase their feeling of uncertainty.

“A doctor told us that [the child] has an aggressive disease but no one ever told me before that. I confronted the nurse coordinator and asked why he didn’t tell me before that he [the child] has an aggressive disease, he said that he did tell me he had and active disease. I told him you didn’t say active or aggressive, you didn’t tell me anything.” (Father of 4 years old child, age 36, Jordanian)

“I: Do you have enough medical information regarding her situation?

CG: Well, no, I don’t think so. I just know that she’s taking chemotherapy and today was supposed to be her 6th cycle but then they told us that she has radiotherapy instead. So today is her first session of radiotherapy [ … ] I like to know everything about my daughter like when she should finish treatment and what happened to the tumor – is it still the same or did it shrink.” (Mother of 3 years old child, age 26, Jordanian).

Some caregivers reported that addressing clinical issues was their priority. The majority of caregivers, more than half of HCPs and half of children stated that HCPs, especially doctors, tended to focus more on clinical information and they considered it was not their job to share or interact with non-clinical issues.

“I think physical issues are the most important. If a symptom is upsetting her, they should check that first. Psychological issues are not really their thing, [ … ] as doctors, they should mainly think about the body, and how different medications are affecting it, or if it is causing any side effects. This is a hospital, not a chapel.” (Mother of 16 years old child, age 46, Jordanian)

However, some parents and children reported that sharing non-clinical information could help them to vent and make the HCPs understand their needs better which would be reflected on the child’s health status.

“It is important to ask about the mood, ask them about their likes and dislikes, that should be included, what they feel like doing, whether they want to play or to listen to music, or even dance and sing, or maybe go out. The questions you asked me earlier should be included because it makes some people feel better.” (child, age 15, female, Jordanian)

“They should ask about the mental state. If the parents are in a good place mentally, the child will benefit more from treatment.” (Mother of 3 years old child, age 46, Jordanian)

Some HCPs recognized the need of non-clinical information to help in assessing patients’ physical issues, such as pain and insomnia. They shared that some physical symptoms may result from non-clinical issues such as social, emotional and psychological problems.

“Sometimes problems manifest as physical symptoms. Like, if the patients don’t want to eat, it may be due to pain but it can also be because the child saw his/her parents fight or is worried or upset about something and it’s keeping the child up at night. So, not all physical symptoms are cause by a medical pathology, sometimes it’s due to psychological or social factors. For example, a child with divorced parent [ … ] never been given enough attention and they always want the medical team to care for them, so they fake the pain.” (Non-Medical HCP, female, 1 year of experience)

#### 3.2.3 Preferred communication styles

Being compassionate and understanding were mentioned by some HCPs and caregivers as an important aspect to consider when communicating with caregivers and children. This included using compassionate verbal and nonverbal communication with caregivers and children, acknowledging their suffering and feelings, and giving them adequate time to answer their inquiries.

“It’s unnatural the way she holds all of these children, you feel like she is a human in every sense of the word. She gives you enough time, if you forget any questions, she reminds you of them. She’s unreal when you deal with her you wonder why there aren’t more people like this … honestly some doctors if they want to hand you the child it’s like gum. They don’t look at the child or acknowledge him even though it’s a child.” (Mother of a 3 years old child, age 44, Jordanian)

“Interviewer: Are you happy with the way the doctors and nurses communicate?

child: It is perfect.

Interviewer: How so?

child: They make me feel at home. They treat me well.

Interviewer: Do you feel they are compassionate?

Child: Yes” (child, age 17, male, Jordanian)

“But the parents are like those drowning who are holding on to a straw, they want anything that might work. He might think it is ridiculous if it was someone’s son, but when it’s his it’s different. And this is something that as a medical staff we must appreciate, we don’t belittle anyone who says anything. There must always be a sense of respect and dignity for the other person so that the relationship can remain healthy.” (Medical HCP, female, 10 years of experience)

For HCPs, effective verbal and non-verbal communication between the three stakeholders relied on trustful relationships for better interaction, understanding, and information sharing.

“They have to be on the same page as their child, they have to know what is wrong with their child and not to tell their child lies, because if that child lost trust in the parents, they will be living in their own world. Some healthcare professionals, not many in our center, lack experience and cannot use proper words. Choosing words is very essential with children, as many of our children are too aware for their age.” (Medical HCP, female, 3 years of experience)

Some HCPs highlighted the role of experience, time and previously knowing the family and the patient in facilitating effective communication and interactions with them and delivering bad news for individuals.

“He [the child], for example, has been with us for a long time and then dies as we know his family, or we had a relationship with the patient’s family. So, telling them becomes much easier for me. But here is the first time for me, I have known him just for two weeks or a week, and the patient’s family is unknown to me, so telling them becomes much difficult for me, but by the time with the period and with the experience that become a little easier for us.” (Medical HCP, male, 3 years of experience)

Sharing information proactively, before the event, helps in reassuring the family and the patient, decreases their uncertainty and helps them to cope better.

“We all changed, it got better, when you have information about something, you feel relieved, you know what to expect.” (Mother of a 3 years old child, age 35, Jordanian)

“I had spots on my lungs. My parents knew about it and they also didn’t tell me and I found out, there was this guy named [ … ], God bless him, he told me that those who have it in their bone it can come back to their lungs so it might come back for you, so he gave me a hint so I expected it so when I got it I wasn’t shocked” (child, age 15, male, Jordanian)

The findings show that some children and caregivers with limited health literacy need more information and explanation regarding cancer and its related aspects. Some caregivers acknowledged that they would need more explanation to understand things better because they were less educated.

“I want to get the right result. I did not go to school, if you didn’t explain to me what is in this piece of paper, I would never understand a thing even if I stared at it for an entire day.” (Father of a 4 years old child, age 38, Jordanian)

Some HCPs and caregivers mentioned that collaboration and coordination between staff were needed for effective communication as it helped in managing families’ and children’s health-related issues appropriately. Open and regular communication channels were used. Some HCPs reported that they communicated with each other directly face-to-face (e.g., during rounds, clinics, and meetings) or indirectly (e.g. through phone calls, messages, emails, and WhatsApp) as needed.

“We usually see each other during rounds, we see each other in the clinic. If anything happens, we immediately contact each other through the phone and we also have a WhatsApp group. So, we communicate on all levels as they say. So, if one of the parents contacted one of us on duty or someone came during the weekend, she would tell me.” (Medical HCP, female, 10 years of experience)

Some HCPs, caregivers and few children mentioned that the use of different approaches of communication upon the child developmental stage, age group and disease progression were helpful for effective communication between the child and both the staff and caregivers. Searching for alternative ways to communicate with children with disabilities (e.g. losing certain communication abilities such as speech or eyesight) resulting from tumor, even when limited, may address some of these challenges. They reported that using children’s language and aiding material may facilitate communication with the child. Few parents stated that the use of simple and plain language when communicating with young children may make them communicate and understand their health-related information better.

“When Dr [ … ] first explained the illness to me, she did it using Lego with trucks, these things [ … ] like some little kids, like when they come to the center this toy idea, these ideas would really help them.” (child, age 16, male, Jordanian)

“I remember having a patient who lost his sight, could no longer speak, and could no longer hear. They were completely dependent on their sense of touch. The only person they could communicate with was their mother. If he was hungry, he’d press on one finger [ … ] the family’s biggest issue was being able to communicate with their child, which was very difficult to facilitate. What we try to do is create a tool for the neuro-oncology patients so that they aren’t completely incapable of communicating with their family.” (Medical HCP, female, 13 years of experience)

Informing caregivers that disease may cause behavioral changes for the child could help HCPs and caregivers when dealing and communicating with the children. It may also help in preventing conflicts and facilitating communication among the three stakeholders.

“if he [the child] has a brain tumor it changes his behavior. He cannot differentiate if it’s ok to say that or not. So it’s not that he’s impolite, or not to criticize the father, and the behaviors are caused by the tumor. It put pressure on the behavior area, so neither the mother nor the father can let him know what’s right or wrong, especially when the child never said any bad word and all of sudden, he does.” (Medical HCP, female, 15 years of experience)

Some of HCPs and caregivers reported that caregivers may help in communicating and assessing the child’s needs especially younger ones and those who have certain communication limitations. However, these results were mentioned by staff and caregivers only and not by any child.

“If I cannot communicate with the child, I would ask their parents, if they were sleeping, you’ll ask their mom how they’re doing, and whether they have eaten or drunk anything that day. All of this gives an impression.” (Medical HCP, female, 3 years of experience)

“Forget about talking, he can’t even express himself anymore. He just nods his head or blinks for “yes”, he raises his eyebrows for “no” [ … ] we tell them about his symptoms because we can understand him more than the physicians. We’re his parents, we can understand what’s hurting him, like once he was indicating that his leg hurt him by saying “my weg ow”.” (Father of 4 years old child, age 36, Jordanian)

In contrast, a couple of parents verbalized that they found difficulties in understanding their young child’s (5 years old below) complaints and needs, which made them feel frustrated and anxious. They related this to the effect of disease progression or child’s age.

“Little children are tough to understand, and I believe this is the greatest challenge in children younger than five years of age. This is my experience, there were nights when I couldn’t sleep because I was worried, she might be in pain and I couldn’t spot it, so I would keep observing. It was very hard, but it is easier if the child is a bit older.” (Mother of a 3 years old child, age 35, Jordanian)

#### 3.2.4 Communication and information sharing with refugees

Most refugees (caregivers and children) were found to have similar practices, issues, and needs in relation to communication and information sharing themes. For example, caregivers felt they understood their child needs and complains better than HCPs.

“If I ask her [the child] what she feels like eating, I can tell from the way she answers that question if she doesn’t feel like eating. I ask her if I brought her food from home would she eat and she says yes, that means she doesn’t like the food in the hospital and not that she doesn’t feel like eating.” (Grandmother of a 5 years old child, age 55, Palestinian)

Refugee children and caregivers reported facing no discrimination or problems regarding a language/dialect barrier between them and their HCPs or their ability to understand or being understood.

“It wasn’t that difficult, we have similar vocabulary, very little difference. We understand what you say” (Father of 2 years old child, age 45, Libyan).

“Interviewer: Do you face any trouble understanding some of the words the medical team uses? Do you have an issues with the dialect or do you understand clearly? *child: No, I understand.”* (child, age 17, male, Syrian)

In contrast, some HCPs shared that some refugees had difficulties in understanding their dialect, especially the Libyans and Iraqis. Other HCPs stated that they faced problem in understanding some refugees’ dialect words, especially at the beginning when refugees started to come for treatment at the center. However, with time HCPs get more used to different refugees’ dialects. They reported that refugees’ dialect issues may vary between and within cultural contexts especially for Libyan, Iraqi and Yemeni.

“When the Libyans first came, it was hard to understand some of the sentences they were saying. I remember having a Yemeni patient who I didn’t know how to collect history from, it is probably due to the cultural background of the patient. Other Yemeni patients were easy to talk to, but I can’t generalize and say we have a communication issue with them here at KHCC.” (Medical HCP, female, 3 years of experience)

“The Libyans in particular would face great difficulty in interpreting our dialect, sometimes the doctor would come to speak with them, and they can’t understand a thing, so this problem needs a solution. I mean, the Syrian dialect is similar to the Jordanian dialect, but the Libyans and Iraqis aren’t very familiar with it.” (Medical HCP, female, 1 year of experience).

Some of Libyan refugees had misleading information, which created communication challenges for HCPs. Some Libyan caregivers had unrealistically high expectations toward their children’s health status though they came to the center with a progressive terminal stage, and expected their child to be cured. This may result in communicational conflicts between the staff and refugees and parents or/and refugees and non-Jordanian parents’ dissatisfaction.

“I mean, the Libyan patients, do you understand how? I mean, the problem is that they do not come to us at the early stage, they are all in complicated stage and end stage, and their situation is complicated, you understand how? And the problem is that they have been told if they go to Al- Hussein Centre, they will be cured, if God’s will [ … ] I mean, he is coming with a belief, and you are coming to change their belief [ … ] Therefore, dealing with them is a little more difficult.” (Medical HCP, male, 3 years of experience)

## 4 Discussion

This novel study has revealed communication and information sharing preferences and practices as well as generated recommendations for good practice from primary data collected with children, family members and health care professionals in advanced cancer care in Jordan.

Our study demonstrated the importance and feasibility to involve children participants in palliative care research. It highlighted that children also hide information from their parents to protect them, and that HCPs need to ask whether or not they want to be involved in decision-making or to receive information about their treatment or disease.

We found that hiding information was a frequent practice by all stakeholders. These results contradict WHO recommendations for children’s involvement in their health care (28), and the United Nations convention on the rights of child. Furthermore, hiding information from patients may form a moral challenge for HCPs who have to be honest with the patients to gain their trust while respecting families’ wishes (29, 30). This trustful relationship built on honesty, is the cornerstone of effective communication (31). Best practices must reflect the religious beliefs of Muslim-majority countries with respect to end-of-life (32).

Our data showed that sharing and addressing enough non-medical information is crucial to enhance patients’ and caregivers’ satisfaction, high quality patient and family-centered care and improve their mental wellbeing (33; 34). However, HCPs should check patients’ and caregivers’ preferences regarding information sharing. HCPs should re-check children preferences over time as the children mature and may change their preferences and needs (2). Some children may wish to have full information and be involved in their treatment plan, others may choose not to know and to delegate decisions to their parents.

We found that parents and children in Jordan value compassionate, understanding and proactive communication. They wanted their HCPs to collaborate and coordinate between each other to understand families’ suffering and to acknowledge the difficulties they were facing. They wanted to be heard and be given the chance to talk. This concurs with the findings of a systematic review including Eastern and Western studies (2).

Our participants suggested that different approaches of communication could be adapted to fit patients’ age and developmental stage. For example, using toys in our sample worked well in facilitating communication and enhancing child understanding (31; 35; 36).

HCPs working with refugees and refugees may face challenges in communication such as dialect differences and potential unrealistic expectations regarding their child’s treatment and disease progression. This high expectation may have resulted from the good reputation of the treatment center in the surrounding regions. Previous study with caregivers of children with cancer concluded that communication complexity increased greatly with patients and families from conflict-affected areas (13).

### 4.1 Strengths and limitations

To the best of our knowledge, this is the first study in the region to describe the views of children and caregivers and their HCPs regarding communication and information sharing in pediatric palliative cancer care. Involving palliative children with terminal diseases in such studies is limited worldwide, especially in low- and middle-income countries (16). Participating stakeholders provided us the opportunity to have more insightful, in-depth data that helped us to meet our study aim and objectives from different perspectives.

The methodological approach used in this study including a variety of interview topic guides, the qualified experienced interviewers, the collaborative approach between the research team in research conduction and analysis and the regular meetings, resulted in producing high quality data. Additionally, the recruitment of a variety of stakeholders, such as a culturally-diverse research team from different countries with various backgrounds (e.g., nursing, public health, and psychology) as well as the different interpretations in this study’s initial framework gave our research a culturally and contextually broad perspective and guaranteed data credibility.

Regarding our study limitations, our findings may not be generalizable to the whole region because of the relatively small sample size and the fact that we recruited participants from one cancer institution in Jordan. This may result in excluding participants from another institution (selection bias) who may have different perspectives and experiences of pediatric palliative care. Those with poor engagement in care or greatest distress may not have had the opportunity to engage in the research. Furthermore, parents’ religion and socio-economic status were an important issue that has not been addressed in our study. Addressing these aspects may give us a clearer view of parents’ religion and socio-economic status and its effect on communication and information sharing in this particular context.

### 4.2 Contribution to the pediatric palliative cancer care future practice

Communication and information sharing in pediatric palliative care settings is an essential component of care to enhance patients’ and families’ quality of life and enhance the patient-centeredness of care (2, 9, 10). However, concurrent relevant stakeholders’ views (children, families and health care professionals) are rarely collected together, and refugee families have been rarely reported. Our findings emphasize the need to respect patients’ and caregivers’ rights and preferences, to know how and when to share information in line with cultural and educational backgrounds.

We need to improve stakeholders’ (including public) awareness regarding palliative care holistic approach, team roles and services, and the importance of addressing clinical and non-clinical information and issues. Enhanced HCPs training regarding communication and information sharing with children and caregivers in pediatric palliative cancer care settings is required. There is also a need to develop a suitable child-centered outcome measure that assesses child’s and family’s needs and concerns including communication and information sharing. In addition, refugees deserve particular attention and support taking into consideration addressing any issues related to their dialect and child treatment misconceptions.

## 5 Conclusion

Communication and information-sharing is a core aspect of quality cancer care for children with advanced disease. The findings of this study can guide evidence-based, culturally appropriate and acceptable care in advanced disease. Thus would put the child and the family at the center of decision making and support, and enable health care professionals to have confidence in initiating relevant discussions. Conducting a multi-center study in culturally homogeneous settings, focusing on communication and information sharing, is recommended.

## Data availability statement

The original contributions presented in the study are included in the article/supplementary material. Further inquiries can be directed to the corresponding authors.

## Ethics statement

The studies involving human participants were reviewed and approved by Kings College London (Study Reference: HR-18/19-8838) and King Hussein Cancer Centre (Study Reference: 18 KHCC 162). Written informed consent to participate in this study was provided by the participants’ legal guardian/next of kin.

## Author contributions

The study was conceptualized and designed by RH, PG and OS, with input from SA, OS, GA and WA. GA, WA, and SA recruited participants and collected data. GA and WA worked on the interview transcriptions. GA, SB and WA jointly analyzed and interpreted the data. GA wrote the manuscript. All authors contributed to the article and approved the submitted version.

## References

[1] Steliarova-Foucher E Colombet M Ries LAG Moreno F Dolya A Bray F. International incidence of childhood cancer 2001–10: a population-based registry study. Lancet Oncol (2017) 18(6):719–31. doi: 10.1016/S1470-2045(17)30186-9 PMC5461370 28410997

[2] Lin B Gutman T Hanson CS Ju A Manera K Butow P. Communication during childhood cancer: systematic review of patient perspectives. Cancer (2020) 126(4):701–16. doi: 10.1002/cncr.32637 31821552

[3] Howard SC Zaidi A Cao X Weil O Bey P Patte C. The my child matters programme: effect of public–private partnerships on pediatric cancer care in low-income and middle-income countries. Lancet Oncol (2018) 19(5):e252–66. doi: 10.1016/S1470-2045(18)30123-2 29726390

[4] Mojen LK Rassouli M Eshghi P Sari AA Karimooi MH. Palliative Care for Children with Cancer in the Middle East: A Comparative Study. Indian J Palliat Care (2017) 23(4):379–86. Available from: https://pubmed.ncbi.nlm.nih.gov/29123342/. 10.4103/IJPC.IJPC_69_17 PMC5661338 29123342

[5] Sleeman KE Gomes B de Brito M Shamieh O Harding R. The burden of serious health-related suffering among cancer decedents: global projections study to 2060. Palliative Med (2021) 35(1):231–5. doi: 10.1177/0269216320957561 PMC7797611 32945226

[6] Gheorghe A Chalkidou K Shamieh O Kutluk T Fouad F Sultan I. Economics of pediatric cancer in four Eastern Mediterranean countries: a comparative assessment. JCO Glob Oncol (2020) 6:1155–70. doi: 10.1200/GO.20.00041 PMC7392699 32697668

[7] Levine DR Liederbach E Johnson LM Kaye EC Spraker-Perlman H Mandrell B. Are we meeting the informational needs of cancer patients and families? perception of physician communication in pediatric oncology. Cancer Cancer (2019) 125(9):1518–26. doi: 10.1002/CNCR.31937 PMC6945977 30602057

[8] Blazin L Cecchini C Habashy C Kaye E Baker J. Communicating effectively in pediatric cancer care: translating evidence into practice. Children (2018) 5(3):40. doi: 10.3390/children5030040 29534479 PMC5867499

[9] Brédart A Bouleuc C Dolbeault S. Doctor-patient communication and satisfaction with care in oncology. Curr Opin Oncol (2005) 17(4):351–4. doi: 10.1097/01.cco.0000167734.26454.30 15933466

[10] Levetown M. Communicating with children and families: from everyday interactions to skill in conveying distressing information. Pediatrics (2008) 121(5):e1441–60. doi: 10.1542/peds.2008-0565 18450887

[11] Ranmal R Prictor M Jt S Ranmal R Prictor M Jt S. Interventions for improving communication with children and adolescents about their cancer. Cochrane Database Syst Rev (2008) 4:CD002969. doi: 10.1002/14651858.CD002969 PMC11663507 18843635

[12] Coyne I Amory A Gibson F Kiernan G. Information-sharing between healthcare professionals, parents and children with cancer: more than a matter of information exchange. Eur J Cancer Care (2016) 25(1):141–56. doi: 10.1111/ecc.12411 26537295

[13] Atout MM Carter B. Communication styles between family carers and children with leukaemia in occupied Palestinian territory. J Child Health Care (2021) 25(3):427–41. doi: 10.1177/1367493520949318 32783747

[14] Arabiat DH Alqaissi NM Hamdan-Mansour AM. Children’s knowledge of cancer diagnosis and treatment: Jordanian mothers’ perceptions and satisfaction with the process. Int Nurs Rev (2011) 58(4):443–9. doi: 10.1111/j.1466-7657.2011.00899.x 22092322

[15] Omar O Wynaden D. Perceptions of Jordanian children with cancer regarding concealing the true nature of their diagnosis: an interpretive phenomenological analysis study. GJMEDPH (2013) 2(6):1–9.

[16] Namisango E Bristowe K Murtagh FEM Downing J Powell RA Abas M. Towards person-centred quality care for children with life-limiting and life-threatening illness: self-reported symptoms, concerns and priority outcomes from a multi-country qualitative study. Palliative Med (2020) 34(3):319–35. doi: 10.1177/0269216319900137 32081084

[17] Guo P Alajarmeh S Alarja G Alrjoub W Al-Essa A Abusalem L. Compounded trauma: a qualitative study of the challenges for refugees living with advanced cancer. Palliative Med (2021) 35(5):916–26. doi: 10.1177/02692163211000236 PMC8114446 33765877

[18] Abdel-Razeq H Attiga F Mansour A. Cancer care in Jordan. Hematol Oncol Stem Cell Ther (2015) 8(2):64–70. doi: 10.1016/j.hemonc.2015.02.001 25732671

[19] Tong A Sainsbury P Craig J. Consolidated criteria for reporting qualitative research (COREQ): a 32-item checklist for interviews and focus groups. Int J Qual Health Care (2007) 19(6):349–57. doi: 10.1093/intqhc/mzm042 17872937

[20] Coombes LH Wiseman T Lucas G Sangha A Murtagh FE. Health-related quality-of-life outcome measures in paediatric palliative care: a systematic review of psychometric properties and feasibility of use. Palliative Med (2016) 30(10):935–49. doi: 10.1177/0269216316649155 PMC5117129 27247087

[21] Coombes L Bristowe K Ellis-Smith C Aworinde J Fraser LK Downing J. Enhancing validity, reliability and participation in self-reported health outcome measurement for children and young people: a systematic review of recall period, response scale format, and administration modality. Qual Life Res (2021) 30:1803–32. doi: 10.1007/s11136-021-02814-4 PMC8233251 33738710

[22] Coombes L Braybrook D Roach A Scott H Harðardóttir D Bristowe K. Achieving child-centred care for children and young people with life-limiting and life-threatening conditions-a qualitative interview study · on behalf of c-POS. Eur J Pediatr (2022) 1:3. doi: 10.1007/s00431-022-04566-w PMC9371630 35953678

[23] Al-Sheyab NA Alomari MA Khabour OF Shattnawi KK Alzoubi KH. ‘Assent and consent in pediatric and adolescent research: school children’s perspectives’, adolescent health, medicine and therapeutics. Dove Press (2019) 10:7. doi: 10.2147/AHMT.S185553 PMC6375532 30804694

[24] Pask S Pinto C Bristowe K van Vliet L Nicholson C Evans CJ. A framework for complexity in palliative care: a qualitative study with patients, family carers and professionals. Palliative Med (2018) 32(6):1078–90. doi: 10.1177/0269216318757622 29457743

[25] Saunders C. The philosophy of terminal care. Ann Acad Med Singap (1987) 16(1):151–4. 3592584

[26] Gale NK Heath G Cameron E Rashid S Redwood S. Using the framework method for the analysis of qualitative data in multi-disciplinary health research. BMC Med Res Methodol (2013) 13:117. doi: 10.1186/1471-2288-13-117 24047204 PMC3848812

[27] Steliarova-Foucher E Stiller C Lacour B Kaatsch P. International Classification of Childhood Cancer, third edition. Cancer (2005) 103(7):1457–67. doi: 10.1002/cncr.20910 15712273

[28] Organization WH. Integrating palliative care and symptom relief into paediatrics: a WHO guide for health-care planners, implementers and managers. World Health Organization (2018). 87 p.

[29] Aljubran AH. The attitude towards disclosure of bad news to cancer patients in Saudi Arabia. Ann Saudi Med (2010) 30(2):141–4. doi: 10.4103/0256-4947.60520 PMC2855065 20220264

[30] Khalil RB. Attitudes, beliefs and perceptions regarding truth disclosure of cancer-related information in the middle East: a review. Palliat Support Care (2013) 11(1):69–78. doi: 10.1017/S1478951512000107 23171758

[31] Bahrami M Namnabati M Mokarian F Oujian P Arbon P. Information-sharing challenges between adolescents with cancer, their parents and HCPss: a qualitative study. Support Care Cancer (2017) 25(5):1587–96. doi: 10.1007/s00520-016-3561-z 28078477

[32] Abdullah R Guo P Harding R. Preferences and experiences of Muslim patients and their families in Muslim-majority countries for end-of-Life care: a systematic review and thematic analysis. J Pain Symptom Manage (2020) 60(6):1223–1238.e4. doi: 10.1016/j.jpainsymman.2020.06.032 32659320

[33] Wilson-Stronks A Cordero CL Carr M. The joint commission isa Rodriguez, project coordinator, division of quality measurement and research, the joint commission Mara youdelman (2010). National Health Law Program. Available at: http://www.jointcommission.org (Accessed 26 November 2021).

[34] Clay AM Parsh B. Patient- and Family-Centered Care: It’s Not Just for Pediatrics Anymore. AMA J Ethics (2016) 18(1):40–4. Available from: https://pubmed.ncbi.nlm.nih.gov/26854635/. 10.1001/journalofethics.2016.18.1.medu3-1601 26854635

[35] Bell J Condren M. Communication strategies for empowering and protecting children. J Pediatr Pharmacol Ther (2018) 21(2):176–84. 10.5863/1551-6776-21.2.176 PMC4869776 27199626

[36] Utilización L De Niños T Multidisciplinario E. The use of the toy during the treatment of children with cancer: perceptions of the multidisciplinary team. Revista Brasileira de Cancerologia (2018) 64(3):309–15. doi: 10.32635/2176-9745.RBC.2018v64n3.28

